# Epidemiologic and Clinicopathological Characterization of Feline Mammary Lesions

**DOI:** 10.3390/vetsci11110549

**Published:** 2024-11-07

**Authors:** Fernanda R. Souza, Isabella S. Moreira, Artur A. Dariva, Karen Y. R. Nakagaki, Camila C. Abreu, Débora Balabram, Geovanni D. Cassali

**Affiliations:** 1 Department of General Pathology, Institute of Biological Sciences, Federal University of Minas Gerais, Av. Presidente Antônio Carlos, 6627, Belo Horizonte 31270-901, Brazil; fersouza.vet@gmail.com (F.R.S.); isabellasmoreira97@gmail.com (I.S.M.); arturalvesdariva@gmail.com (A.A.D.); karenyumi@ymail.com (K.Y.R.N.); 2 Vale Veterinary Pathology, Av. Voluntário Benedito Sérgio, 1535, Taubate 12053-000, Brazil; camilacabreu@gmail.com; 3 Department of Surgery, Faculty of Medicine, Federal University of Minas Gerais, Av. Prof. Alfredo Balena, 190, Belo Horizonte 30130-100, Brazil; debalabra@gmail.com

**Keywords:** cats, histopathology, mammary tumor, neoplasia, classification, epidemiology, diagnosis, oncology

## Abstract

Most lesions found in the mammary glands of cats are malignant, with aggressive behavior and unfavorable prognosis. Studies on the epidemiologic and clinicopathological characteristics of mammary lesions in cats are scarce. The present study aimed to evaluate those characteristics and to correlate them with survival in cats. Mammary specimens were selected from 418 domestic cats that underwent surgical removal with or without lymphadenectomy. The cats and mammary lesions were evaluated for epidemiologic, clinical, and pathologic characteristics. Cats with malignant neoplasms were older than cats with benign neoplasms and non-neoplastic lesions; 858 lesions were identified, including sporotrichosis, basaloid carcinoma, and benign phyllodes, described for the first time in cats. Tubulopapillary and cribriform carcinomas were the most common malignant tumors found and were very similar in characteristics such as marked anisocytosis/anisokaryosis, high mitotic count (score 3) (*p* < 0.001), and presence of necrosis (*p* = 0.005). The association between advanced age and malignancy, as well as the description of new lesions, emphasizes the importance of population studies in cats to understand the behavior of the disease and to draw attention to diagnoses that should be considered in routine care.

## Simple Summary

Mammary neoplasms are common in intact domestic cats. These tumors are often malignant and metastatic, resulting in unfavorable clinical outcomes for affected animals. Currently, there are few studies that have evaluated the clinical, epidemiologic, and pathologic characteristics of mammary tumors in cats. The aim of this study was to evaluate these characteristics. Our research is relevant because we obtained one of the largest numbers of animals evaluated, found mammary lesions not previously described in feline species, and observed the malignant behavior of histologic types frequently found in routine clinical pathology.

## 1. Introduction

Mammary neoplasms are the third most common tumor type in felines (*Felis silvestris catus*), following hematopoietic and cutaneous neoplasms [1,2,3,4]. A study conducted in the southern region of Brazil revealed that mammary tumors represent the second most prevalent type of neoplasms observed in domestic felines subjected to biopsy and necropsy [5]. However, the discrepancy in incidence between countries is attributed to the implementation of neutering policies [6]. Mammary neoplasms in cats are often malignant, representing between 85 and 95% of diagnoses. These neoplasms tend to be aggressive, and lymph node metastases are more common at the time of diagnosis than in dogs [7,8].

Female cats are susceptible to developing mammary tumors as a result of exposure to ovarian hormones [8]. In comparison to cats that have been neutered, unspayed queens have a sevenfold increased risk of developing tumors [1]. This risk is also due to progestin administration [9]. The average age at which cats develop malignant mammary neoplasms is between 10 and 12 years [1,6,8].

There are contradictions regarding breed predisposition. Siamese cats have a higher risk of developing mammary tumors compared to other breeds, with an average age at onset of 9 years [2,8]. However, domestic shorthaired (DHS) cats have the largest population in the world, reflecting the high incidence of neoplasia in this breed [6]. In Brazil, no breed predisposition has been identified, as the majority of the country’s domestic cat population consists of crossbreeds [10]. In male cats, the occurrence is considered rare and late, affecting animals with an average age of 12.8 years [11].

A retrospective study was conducted to evaluate the outcomes of tumor resection in 107 cats. Median progression-free survival was longer in cats that underwent bilateral mastectomy compared to those that underwent unilateral mastectomy [12]. Bilateral mastectomy is the recommended treatment for mammary neoplasms in cats [8]. Despite this, surgical excision is often non-curative due to the difficulty of completely removing the tumor, particularly in cases where ulceration and invasion are present [13,14]. A definitive diagnosis is made through histopathologic examination [15]. Several classifications have been proposed for feline mammary tumors [7,16,17].

Some prognostic factors have already been established for mammary neoplasms in cats [18]. However, while some authors report the lack of prognostic value associated with histological subtypes [7], a study evaluating cats with micropapillary carcinomas demonstrated reduced overall survival (OS) compared to animals with other types of lesions [19]. Similarly, cats with tubulopapillary and complex carcinomas exhibit superior OS compared to those with solid carcinomas [20].

A growing number of animal owners have expressed a desire for additional tools that can enhance the quality of life and survival of their companion animals [14]. The existing literature on the epidemiological and clinicopathological aspects of feline mammary gland neoplasms is comparatively limited in comparison to that of dogs [2,3,4,21]. Therefore, this study aimed to describe the epidemiologic and clinicopathological information of cats with lesions in the mammary glands.

## 2. Materials and Methods

Case selection: A retrospective study was conducted between the years 2007 and 2023, including mammary gland samples from cats that underwent surgical excision, with or without lymphadenectomy. Animals without histopathologically confirmed mammary lesions were excluded. The samples were selected from the archives of three veterinary diagnostic laboratories, LPC and Celulavet in Belo Horizonte, MG, and Patologia Veterinária do Vale in Taubaté, SP. Clinical and epidemiological data, including sex, age, breed, tumor location, lesion side, and surgical techniques, were collected from the medical records. Tumor size (T) and regional lymph nodes (N) were evaluated [17]. Data on distant metastasis (M) and reproductive status were not included because they were not always available. This study was approved by the Ethics Committee of Animal Use (CEUA/UFMG), protocol number 188/2022.

Histopathological analyses: Mammary gland and lymph node specimens were fixed in 10% neutral buffered formalin, embedded in paraffin, sectioned at 4-μm, and stained with hematoxylin and eosin (H&E). Three veterinary pathologists (GDC, KYRN, and CCA) diagnosed all cases, and two veterinary pathologists (GDC and FRS) reviewed them for histopathologic features. Lesions were classified as malignant neoplasms (MN), benign neoplasms (BN), or non-neoplastic lesions (NNL) [17]. For queens with multiple masses, the tumor with the greatest number of malignant features was analyzed. The Nottingham grading system was used for invasive carcinoma grading [22]. In MN, gross morphology was evaluated for ulceration and cystic spaces. Microscopic features, such as anisocytosis, anisokaryosis, mitotic count [23], necrosis, ulceration, lymphovascular invasion, and resection margin, were evaluated. Neoplasms were classified as “pure” if they consisted of a single histological type or “combined” if they contained more than one. The final diagnosis was determined on the predominant pattern.

Immunohistochemical and histochemical analyses: For definitive diagnosis, immunohistochemistry was performed on malignant adenomyoepithelioma cases. Four-micrometer sections were prepared, and a peroxidase system with a secondary antibody was used, detected by an anti-mouse/anti-rabbit system (Novolink Polymer Detection System; Leica Biosystems, Newcastle Upon Tyne, UK). Antigen retrieval was performed in citrate buffer (pH 6.0) in a water bath. Endogenous peroxidase activity was blocked using 10% hydrogen peroxide in methyl alcohol. Mammary gland sections were incubated overnight with the primary antibody p63 (clone DAK-p63, 1:100, Dako) at 4 °C in a humidity chamber. Reagents were applied manually, and immunoreactivity was visualized with diaminobenzidine chromogen (DAB substrate system; Dako) for 3 min. Feline mammary glands were used as positive controls. Negative controls consisted of PBS as a substitute for the primary antibody. Histochemical staining with periodic acid–Schiff (PAS) and Alcian blue (pH 2.7) was performed to confirm mucinous carcinoma.

Data analysis: Statistical analyses were performed using MedCalc (version 20, MedCalc Software Ltd., Ostend, Belgium) and Jamovi (version 2.5) software. The chi-square test was employed to assess the correlation between general variables, including sex, age, breed, tumor localization, lesion side, surgical technique, and tumor size, with the broader diagnostic categories (malignant neoplasm, benign neoplasm, and non-neoplastic lesions). The chi-square test was also applied to analyze the association between gross morphology, microscopic features, and specific malignant histologic types. The features were analyzed with the most common malignant histologic types: tubulopapillary carcinoma, cribriform carcinoma, and malignant adenomyoepithelioma. This entailed comparing gross and microscopic ulceration, cystic spaces, necrosis, lymphovascular invasion, resection margins, anisocytosis, anisokaryosis, and mitotic counts. Values were considered significant when *p* ≤ 0.05.

## 3. Results

### 3.1. Clinical and Epidemiological Characteristics

A total of 418 cats were included. Considering one diagnosis per animal, cats with malignant neoplasms represented 88.8% (371/418), 2.2% (9/418) had benign neoplasms, and 9.1% (38/418) had non-neoplastic lesions. Regarding the epidemiological data, only two cats were male (2/418; 0.5%). Queens with MN were significantly older, with a mean age of 10.2 ± 3.4 years, while female cats with BN had a mean age of 7.0 ± 5.4 years. Cats with NNL were younger, with a mean age of 5.3 ± 4.2 years (*p* < 0.001). Most of the cats were crossbreeds (292/418; 69.9%). Table 1 provides detailed epidemiologic and clinicopathological information for the lesions studied.

### 3.2. Frequency and Characterization of Mammary Lesions and Lymph Node Metastases

Considering all mammary glands examined, 858 lesions were identified, including benign non-neoplastic lesions, malignant neoplasms, and benign neoplasms. The majority were MN with 68.9% (591/858), followed by NNL with 24.7% (212/858) and BN with 6.4% (55/858). All findings are detailed in Table 2.

**Figure 1:**
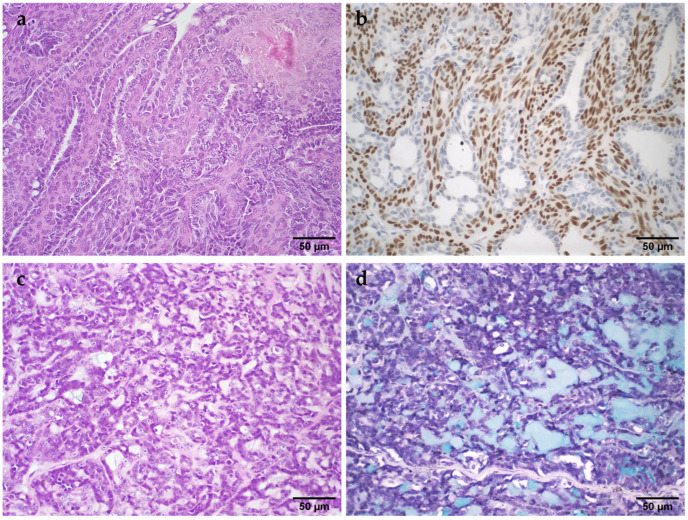
Histopathological images of malignant neoplasms of the mammary gland in female cats. (**a**): Malignant adenomyoepithelioma. The proliferation of myoepithelial cells is observed in the internal region, and epithelial cells are in the peripheral region of the papillary projections, H&E; (**b**): Malignant adenomyoepithelioma. p63-positive myoepithelial cells in the inner region of the papillary projections; (**c**): Mucinous carcinoma. Epithelial cells are observed in association with a slightly basophilic amorphous material (mucin), H&E; (**d**): Mucinous carcinoma. Mucin was positive by the Alcian blue staining technique.

The UDH were associated with MN in 52% of cases. The diagnosis of cases of malignant adenomyoepithelioma and mucinous carcinoma was possible by anti-p63 immunohistochemistry and PAS or Alcian blue staining, respectively (Figure 1).

Common findings included tumor-associated ectasia, concretions (*corpora amylacea*), and inflammatory cell infiltrates of variable distribution and intensity but with a predominance of lymphocytes and plasma cells. Only animals that underwent lymphadenectomy were included for analysis of lymph nodes, representing 53.8% (225/418) of cases. In animals with MN, 49.2% (98/199) had metastases in at least one lymph node.

### 3.3. Analysis of Common Malignant Neoplasms for Macroscopic and Microscopic Features

Considering the most common MN of the feline mammary gland (malignant adenomyoepithelioma, cribriform carcinoma, and tubulopapillary carcinoma), most did not show ulceration or cystic formation on macroscopic examination. Microscopically, there were no significant differences between the three histologic types in relation to ulceration (*p* = 0.085). There was a predominance of clean margins and an absence of lymphovascular invasion in the three groups (*p* < 0.001). There was a significant difference in the characteristics of anisocytosis (*p* < 0.001), anisokaryosis (*p* < 0.001), mitotic count (*p* < 0.001), and ulceration (*p* = 0.005) between malignant adenomyoepitheliomas and cribriform and tubulopapillary carcinomas. In malignant adenomyoepitheliomas, moderate anisocytosis and anisokaryosis were observed, with a predominance of low mitotic counts and an absence of ulceration. In contrast, both cribriform and tubulopapillary carcinomas showed marked anisocytosis and anisokaryosis, high mitotic counts, and ulceration (Table 3).

### 3.4. Identification and Histologic Characterization of Novel Lesions in the Feline Mammary Gland

- Sporotrichosis

**Figure 2:**
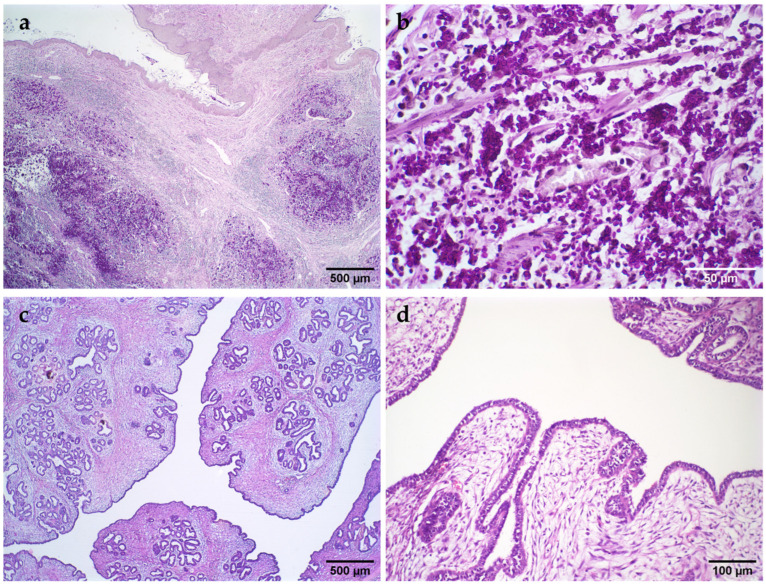
Histopathological images of lesion of the mammary gland in female cats. Sporotrichosis. (**a**): Mammary papilla (upper part of the image). Multiple foci of densely stained inflammatory infiltrates are observed, PAS, 40× magnification; (**b**): Multiple oval and round structures (compatible with *Sporothrix* sp.) stained magenta, PAS; Benign phyllodes tumor (**c**): Slit-shaped lumens conferred foliate appearance, H&E; (**d**): Epithelial cells lining the stroma, H&E.

Among the NNL, a case of feline sporotrichosis involving the mammary gland (A2) and inguinal lymph node was identified in a 1-year-old crossbreed female. Histology showed an abundance of epithelioid macrophages, intact and degenerated neutrophils, and a discrete amount of lymphocytes and plasma cells in the dermis and mammary gland. In the cytoplasm of the macrophages and occasionally free in the interstitial, there were round, oval, and elongated structures between 4 and 6 µm in diameter, morphologically compatible with *Sporothrix* sp. In addition, a similar lesion was observed in the inguinal lymph node. The diagnosis was confirmed by PAS staining, which demonstrated the fungal structures (Figure 2a,b).

- Benign phyllodes tumor

Three cases of benign phyllodes tumor were identified, characterized by slit-shaped lumens giving a foliate appearance. It has a low mitotic index with discrete to moderate cellularity. Well-differentiated vascular neoformation (pseudoangiomatous stromal hyperplasia—PSH) is often observed (Figure 2c,d).

- Basaloid carcinoma

In the MN, six cases of basaloid carcinoma were identified, characterized by neoplastic formation of epithelial cells arranged in solid nests varying in shape and size and delimited by thin stroma. The tumor cells had sparse cytoplasm, with the nuclei of the centrally located cells appearing slightly pale, while the hyperchromatic nuclei of the peripheral cells were arranged in a palisade pattern.

## 4. Discussion

Few studies have evaluated the epidemiologic and clinicopathological data of mammary neoplasms in domestic cats similarly to the present study [2,3,4,21]. To the authors’ knowledge, the present study represents the largest cohort (n = 418) of these. Consistent with the literature, a predominance of cats with MN was observed [6,8], and these were older, with a mean age of 10.2 ± 3.4 years. Statistically significant differences in age and lesion type were observed. Studies of mammary tumors in domestic cats have also shown a higher incidence of this type of neoplasm in animals between 10 and 12 years of age [1,6,8]. Queens with NNL were younger (5.3 ± 4.2 years). This is probably due to the cases of fibroadenomatous hyperplasia affecting young female cats of reproductive age [6,14,24]. Most of the cats were crossbreeds. The demographic composition of the domestic cat population in Brazil consists mainly of animals that are crossbreeds [10].

The incidence of male cats with neoplasia of the mammary gland is between 1% and 5% and is more common in older animals [6,11]. In the present study, two male cats were affected by mammary tumors, and they were 12 and 18 years old. Consistent with the literature, multiple mammary involvement was common [8]. Although radical mastectomy (unilateral or bilateral) is recommended to reduce the recurrence of tumors [17], only cats with MN and NNL frequently underwent this type of surgery. Queens with BN had more conservative approaches, such as nodulectomy/lumpectomy and regional mastectomy. Most cats had large tumors (T3 > 3 cm). Although tumor size is known to be a prognostic factor [20,25], in this study, we did not find a significant association between this variable and overall survival time.

Studies carried out in Brazil using the World Health Organization (WHO) classification [7] found different frequencies of MN [3,26]. In contrast, the most common neoplasms found in the present study were tubulopapillary, cribriform carcinomas, and adenomyoepithelioma. However, we also observed the combination of more than one histologic type [26]. Consistent with the literature, BN was uncommon [6]. Among NNL, fibroadenomatous hyperplasia is the most common lesion [27]. In our study, this was the third most common histologic type of NNL after mastitis and UDH. Mastitis is considered rare in female cats [28]. A possible explanation for the different results between the frequencies of histological types may be the difference in the classifications used and in the sample populations. The presence of UDH in cats was described [29] and compared with those found in women, where 47% of cases were associated with malignancy. Similarly, we found that 52% of UDH were associated with other MN. However, there is not enough literature to provide information on the prevalence of mastitis and UDH in the feline species.

To the authors’ knowledge, this is the first case of feline sporotrichosis involving the mammary gland reported in the literature. This highlights the importance of accurate diagnosis, as not all mammary lesions are tumors. Neoplasms were also described for the first time in cats, such as cases of benign phyllodes and basaloid carcinoma, according to histologic criteria established for women [30] and female dogs [31]. As observed in the present study, ductal ectasia, *corpora amylacea*, and stromal inflammation are common findings [32].

Concerning the three most frequent MN in the studied population, when comparing the malignant adenomyoepitheliomas with the carcinomas (tubulopapillary and cribriform) in terms of histopathologic features, the differences in necrosis, anisocytosis, anisokaryosis, and mitotic count were statistically significant. These features are considered criteria for malignancy [18]. Malignant adenomyoepithelioma showed predominance of moderate anisocytosis and anisokaryosis, an absence of necrosis, and low mitotic count (score 1). Tubulopapillary and cribriform carcinomas showed a higher frequency of marked anisocytosis and anisokaryosis, the presence of necrosis, and high mitotic count (score 3). Although the results suggest that malignant adenomyoepitheliomas may exhibit a less aggressive behavior when compared to carcinomas, it is important to note that we are not able to prove this definitively on the basis of the current data.

Malignant adenomyoepithelioma is a neoplasm that is poorly described in cats, with a small number of cases [33,34]. In addition to morphologic features, immunohistochemistry for p63 allows the identification of the myoepithelial component in this type of neoplasia [34,35]. The clinical course of this type of neoplasia is unknown. Therefore, there is a need for studies that can follow animals diagnosed with malignant adenomyoepithelioma to evaluate the behavior of this histological type. Similarly, to our knowledge, no study has exclusively evaluated cribriform carcinomas for macroscopic and histopathologic characteristics. As for tubulopapillary carcinomas, mitotic figures were frequently observed in a case with spindle cell metaplasia in a cat [36]. Further studies with a larger number of samples, complete data, and animal follow-up are needed to evaluate the clinical behavior of these neoplasms.

It is important to consider the limitations of this study. As this was a retrospective study, there was a paucity of data in the medical records, including details of distant metastasis, reproductive status, recurrence, and chemotherapy. In this case, the unavailable data outnumbered those available. Furthermore, obtaining information on the follow-up of the animals proved to be challenging. Many animals were not taken to the clinics after surgery, resulting in a loss of information. These data are crucial for assessing the prognostic value in cases of mammary neoplasm in felines.

## 5. Conclusions

This study is a retrospective approach to mammary lesions in domestic cats, performing clinicopathological and epidemiologic characterization. Older cats are predisposed to develop malignant neoplasms, so early detection of this type of tumor in older animals is important. In addition, differential diagnoses such as sporotrichosis, an infectious disease that can affect the mammary glands, should be considered. The tumors were described for the first time in feline species, contributing to the knowledge of mammary neoplasms in cats. Malignant neoplasms should be studied separately because the biological behavior may be different between different types of malignant tumors, as in the case of adenomyoepitheliomas, which have characteristics of less malignancy when compared to carcinomas. Cases with complete clinicoepidemiological information and monitoring of animal survival may help in the development of prognostic tools for feline mammary tumors.

## Figures and Tables

**Table 1:** Epidemiological and clinical information for malignant neoplasms, benign neoplasms, and non-neoplastic mammary gland lesions in cats (*n* = 418).

	Malignant Neoplasm (MN)	Benign Neoplasms (BN)	Non-Neoplastic Lesions (NNL)	*p* Value
Sex				
Female	369 (99.5%)	9 (100%)	38 (100%)	0.88
Male	2 (0.5%)	0 (0.0%)	0 (0.0%)	
Total	371	9	38	
Age				
Kitten (1 year or less)	3 (0.8%)	2 (22.2%)	8 (21.1%)	<0.001
Young adult (1–6 years)	34 (9.2%)	1 (11.1%)	11 (28.9%)	
Mature adult (7–10 years)	128 (34.6%)	1 (11.1%)	6 (15.8%)	
Senior (>10 years)	137 (37.0%)	2 (22.2%)	4 (10.5%)	
Total	302	6	29	
Breed				
Purebred	69 (18.6%)	0 (0.0%)	6 (15.8%)	0.38
Crossbreed	260 (70.1%)	7 (77.8%)	25 (65.8%)	
Total	329	7	31	
Tumor location *				
T1	25 (6.7%)	0 (0.0%)	3 (7.9%)	0.07
T2	30 (8.1%)	0 (0.0%)	1 (2.6%)	
A1	34 (9.2%)	3 (33.3%)	5 (13.2%)	
A2	50 (13.5%)	1 (11.1%)	1 (2.6%)	
Multicenter	103 (27.8%)	2 (22.2%)	7 (18.4%)	
Total	242	6	17	
Side of the lesion				
Right	82 (22.1%)	4 (44.4%)	10 (26.3%)	0.18
Left	95 (25.6%)	2 (22.2%)	3 (7.9%)	
Bilateral	55 (14.8%)	0 (0.0%)	4 (10.5%)	
Total	232	6	17	
Surgical technique				
Nodulectomy/Lumpectomy	54 (14.6%)	3 (33.3%)	11 (28.9%)	0.37
Simple mastectomy	84 (22.6%)	1 (11.1%)	8 (21.1%)	
Regional mastectomy	47 (12.7%)	3 (33.3%)	3 (7.9%)	
Combined	8 (2.2%)	0 (0.0%)	0 (0.0%)	
Total	25 (6.5%)	0 (0.0%)	2 (5.3%)	
Tumor size	218	7	24	
T1 (<2 cm)	123 (33.2%)	2 (22.2%)	14 (36.8%)	0.08
T2 (2–3 cm)	101 (27.2%)	2 (22.2%)	2 (5.3%)	
T3 (>3 cm)	133 (35.8%)	5 (55.6%)	20 (52.6%)	
Total	357	9	36	

* T, thoracic mammary gland; A, abdominal mammary gland.

**Table 2:** Histologic classification and frequency of mammary lesions in female cats.

Groups	Histological Classification	n	%
Malignant neoplasms n = 591	Tubulopapillary carcinoma	147	17.1%
	Cribriform carcinoma	144	16.8%
	Malignant adenomyoepithelioma	104	12.1%
	Carcinoma in situ	45	5.2%
	Tubular carcinoma	42	4.9%
	Papillary carcinoma (invasive and noninvasive)	32	3.7%
	Solid papillary carcinoma	11	1.3%
	Solid carcinoma	11	1.3%
	Mucinous carcinoma	10	1.2%
	Micropapillary carcinoma	9	1.0%
	Basaloid carcinoma	6	0.7%
	Papilloma with ductal carcinoma in situ	6	0.7%
	Carcinoma with solid pattern	6	0.7%
	Carcinoma in a mixed tumor	6	0.7%
	Apocrine carcinoma	4	0.5%
	Neuroendocrine carcinoma	2	0.2%
	Secretory carcinoma	2	0.2%
	Carcinosarcoma	2	0.2%
	Carcinoma with sebaceous differentiation	1	0.1%
	Lipid-rich carcinoma	1	0.1%
Non-neoplastic lesions n = 212	Mastitis	69	8.0%
	Usual ductal hyperplasia (UDH)	62	7.2%
	Fibroadenomatous hyperplasia (fibroepithelial hyperplasia)	39	4.5%
	Columnar cell alteration	16	1.9%
	Duct ectasia	11	1.3%
	Adenosis	4	0.5%
	Mastitis obliterans	4	0.5%
	Atypical ductal hyperplasia (ADH)	3	0.3%
	Lobular hyperplasia	3	0.3%
	Sporotrichosis (*Sporothrix* sp.)	1	0.1%
Benign neoplasms n = 55	Adenoma (tubular/ductal/basaloid)	28	3.3%
	Ductal papiloma	16	1.9%
	Benign adenomyoepithelioma	4	0.5%
	Benign phyllodes tumor	3	0.3%
	Sclerosing papilloma	2	0.2%
	Fibroadenoma	1	0.1%
	Benign mixed tumor	1	0.1%
Total		858	100%

**Table 3:** Gross and microscopic characteristics of the main MN of the female cat mammary gland.

	Tubulopapillary Carcinoma	Cribriform Carcinoma	Malignant Adenomyoepithelioma	*p* Value
Ulceration (Gross morphology)				
Present	30 (21.0%)	28 (20.3%)	13 (12.7%)	0.211
Absent	113 (79.0%)	110 (79.7%)	89 (87.3%)	
Cystic spaces (Gross morphology)				
Present	36 (25.2%)	36 (26.1%)	27 (26.5%)	0.971
Absent	107 (74.8%)	102 (73.9%)	75 (73.5%)	
Necrosis				
Present	85 (59.4%)	81 (58.7%)	42 (40.4%)	0.005
Absent	58 (40.6%)	57 (41.3%)	62 (59.6%)	
Ulceration				
Present	26 (18.2%)	24 (17.4%)	9 (8.7%)	0.085
Absent	117 (81.8%)	114 (82.6%)	95 (91.3%)	
Lymphovascular invasion				
Present	32 (22.4%)	42 (30.4%)	6 (5.8%)	<0.001
Absent	111 (77.6%)	96 (69.6%)	98 (94.2%)	
Resection Margins				
Clean	54 (37.8%)	56 (40.6%)	76 (73.1%)	<0.001
Infiltrated	40 (28.0%)	29 (21.0%)	12 (11.5%)	
Close	49 (34.3%)	53 (38.4%)	16 (15.4%)	
Anisocytosis				
Mild	2 (1.4%)	0 (0.0%)	6 (5.8%)	<0.001
Moderate	70 (49.0%)	60 (43.5%)	65 (63.1%)	
Marked	71 (49.7%)	78 (56.5%)	32 (31.1%)	
Anisokaryosis				
Mild	2 (1.4%)	0 (0.0%)	6 (5.8%)	<0.001
Moderate	70 (49.0%)	60 (43.5%)	64 (62.1%)	
Marked	71 (49.7%)	78 (56.5%)	33 (32.0%)	
Mitotic counts				
Score 1 (0–7)	33 (23.1%)	12 (8.7%)	57 (54.8%)	
Score 2 (8–16)	51 (35.7%)	36 (26.1%)	29 (27.9%)	<0.001
Score 3 (>16)	59 (41.3%)	90 (65.2%)	18 (17.3%)	

## Data Availability

The original contributions presented in the study are included in the article; further inquiries can be directed to the corresponding author.
